# Effects of sodium chloride on the gene expression profile of periodontal ligament fibroblasts during tensile strain

**DOI:** 10.1007/s00056-020-00232-8

**Published:** 2020-07-06

**Authors:** Agnes Schröder, Joshua Gubernator, Ute Nazet, Gerrit Spanier, Jonathan Jantsch, Peter Proff, Christian Kirschneck

**Affiliations:** 1 Department of Orthodontics, University Hospital Regensburg, Franz-Josef-Strauß-Allee 11, 93053 Regensburg, Germany; 2 Department of Cranio-Maxillo-Facial Surgery, University Hospital Regensburg, Franz-Josef-Strauß-Allee 11, 93053 Regensburg, Germany; 3 Institute of Clinical Microbiology and Hygiene, University Hospital Regensburg, Franz-Josef-Strauß-Allee 11, 93053 Regensburg, Germany

**Keywords:** Periodontal ligament fibroblast, Periodontal ligament (PDL), Orthodontic tooth movement, Tensile strain, Expression kinetics, Parodontalligament (PDL), Parodontalligament-Fibroblasten, Kieferorthopädische Zahnbewegung, Dehnung, Genexpression

## Abstract

**Purpose:**

During orthodontic tooth movement, pressure and tension zones develop in the periodontal ligament, and periodontal ligament fibroblasts (PDLF) become exposed to mechanical strain. Enhanced salt (NaCl) concentrations are known to modulate responses of PDLF and immune cells to different stimuli like mechanical strain. Here, we investigated the impact of tensile strain on the gene expression profile of PDLF under normal (NS) and high salt (HS) conditions.

**Methods:**

After preincubation under NS or HS (+40 mM NaCl in medium) conditions for 24 h, PDLF were stretched 16% for 48 h using custom-made spherical cap silicone stamps using an established and published setup. After determination of cell number and cytotoxicity, we analyzed expression of genes involved in extracellular matrix reorganization, angiogenesis, bone remodeling, and inflammation by quantitative real-time polymerase chain reaction (RT-qPCR).

**Results:**

Tensile strain did not affect the expression of genes involved in angiogenesis or extracellular matrix reorganization by PDLF, which however modulate inflammatory responses and bone remodeling in reaction to 16% static tensile strain. Salt (NaCl) treatment triggered enhanced extracellular matrix formation, expression of cyclooxygenase 2 and bone metabolism in PDLF during tensile strain.

**Conclusions:**

Salt (NaCl) consumption may influence orthodontic tooth movement and periodontal bone loss via modulation of extracellular matrix and bone metabolism. Excessive salt intake during orthodontic therapy may cause adverse effects regarding periodontal inflammation and bone resorption.

## Abstract (de)

**Ziel:**

Während der kieferorthopädischen Zahnbewegung entwickeln sich Druck- und Zugzonen im Parodontalligament, und parodontale Ligamentfibroblasten (PDLF) werden mechanischer Belastung ausgesetzt. Es ist bekannt, dass erhöhte Salzkonzentrationen (NaCl) die Reaktionen von PDLF und Immunzellen auf verschiedene Stimuli wie mechanische Beanspruchung modulieren. Hier untersuchten wir den Einfluss von Dehnung auf das Genexpressionsprofil von PDLF unter Normal- (NS) und unter Hochsalzbedingungen (HS).

**Methoden:**

Nach 24 h Vorinkubation unter NS- bzw. HS-Bedingungen (+40 mM NaCl im Medium) wurden PDLF 48 h lang unter Verwendung von Silikonstempeln unter Verwendung eines etablierten und veröffentlichten Protokolls um 16 % gestreckt. Nach Bestimmung der Zellzahl und der Zytotoxizität analysierten wir durch RT-qPCR („quantitative real-time polymerase chain reaction“) die Expression von Genen, die an der Reorganisation der extrazellulären Matrix, der Angiogenese, dem Knochenumbau und der Entzündung beteiligt sind.

**Ergebnisse:**

Dehnung hatte keinen Einfluss auf die Expression von Genen, die an der Angiogenese oder der Reorganisation der extrazellulären Matrix durch PDLF beteiligt sind. In Reaktion auf statische Dehnung modulierten PDLF jedoch die Expression von Genen, die an Entzündungsreaktionen und am Knochenumbau beteiligt sind. Eine Behandlung mit Salz (NaCl) bewirkte während der Zugbelastung eine vermehrte Bildung von extrazellulärer Matrix sowie eine verstärkte Expression von Cyclooxygenase 2 und von Markern des Knochenmetabolismus durch PDLF.

**Schlussfolgerungen:**

Salzkonsum kann die kieferorthopädische Zahnbewegung und den parodontalen Knochenverlust durch Modulation der extrazellulären Matrix und des Knochenstoffwechsels beeinflussen. Eine übermäßige Salzaufnahme während der kieferorthopädischen Therapie könnte daher nachteilige Auswirkungen auf parodontale Entzündungen und die Knochenresorption haben.

## Introduction

During orthodontic tooth movement, pressure and tension zones develop in the periodontal ligament (PDL), a connective tissue responsible for the attachment of the tooth to the alveolar bone [25]. Periodontal ligament fibroblasts (PDLF) make up the majority of cells within the periodontal ligament and are the first cells exposed to mechanical stimuli occurring during orthodontic tooth movement. The main function of PDLF is the maintenance of tissue homeostasis and production of collagenous structural proteins and glycosaminoglycans. Furthermore, they sustain regulatory functions in innate immune defense [16, 25].

After an orthodontic force is applied to the tooth, PDLF are subjected to mechanical strain and play an important regulatory role in orthodontic tooth movement. As an early response to compressive forces, PDLF increase prostaglandin production via enhancement of cyclooxygenase 2 (COX-2) activity, resulting in enhanced expression of RANKL (receptor activator of NF-κB ligand) [16, 38, 48], while simultaneously secretion of RANKL decoy receptor osteoprotegerin (OPG) is reduced [38]. This promotes osteoclastogenesis in pressure areas of the PDL resulting in alveolar bone resorption. In contrast, in tension areas bone formation by osteoblastic activity is promoted [25].

Malocclusions have a negative impact on oral-health-related quality of life and mental well-being of children leading to reduced self-confidence [8, 42]. Often these psychological effects triggered by malocclusions are the reason to improve esthetics through orthodontic treatment [42]. Therapy of malocclusions is of distinct medical importance, as recent studies associated malpositioned teeth with the development of caries or periodontitis [2, 37, 41]. Therefore, orthodontic corrections are helpful to prevent the development and progression of these oral diseases. Despite the importance of orthodontic treatment for oral health, many aspects of orthodontic therapy remain uninvestigated, and many problems that arise during an ongoing orthodontic therapy are still unresolved.

Possible influences of nutrition on orthodontic treatment have hardly been investigated. Since orthodontic tooth movement comprises a local sterile-inflammatory process, numerous possibilities exist for the immune system and general metabolism to modulate these processes [18, 53]. Components of nutrition are reported to influence not only chronic diseases like hypertension [22, 23] or osteopenia [44], but also have an impact on the oral microflora [52] and periodontal bone loss [29]. In Western societies electrolytes such as sodium are consumed to a high degree as food supplements in the form of salt (sodium chloride, NaCl) and are known to modulate tissue response to different stimuli by their local tissue concentration [28]. It can be assumed that about 70–80% of the salt intake comes from “hidden” salts in processed foods such as cheese, bread, and ready-to-eat meals [6]. Most people in Europe consume significantly more salt (8–11 g/day) than recommended by the German Nutrition Society (1.5 g/day) [6, 51]. Therefore, it can be assumed that salt-related effects on orthodontic tooth movement in industrialized nations are relevant to the vast majority of patients.

Consumption of high salt diet or inflammation induces Na^+^ accumulation in various tissues, thereby, modulating the activity of cells of the mononuclear phagocytic system [3, 14, 22, 23]. Furthermore, Na^+^ accumulation may also occur in tissues of the oral cavity, especially the gingiva, the oral mucous membrane, and the periodontal ligament [39]. As salt (sodium chloride) is known to have a direct impact on the activity of cells of the mononuclear phagocytic system [14, 23, 32] and osteoclasts [54], it may also have an effect on the response of PDLF to tensile strain.

## Materials and methods

### In vitro cell culture experiments

All experiments were performed in accordance with the relevant guidelines and regulations. Approval for the collection and use of PDLF was obtained from the ethics committee of the University of Regensburg, Germany (approval number 12-170-0150). We obtained informed consent from all patients or their legal guardians.

Periodontal ligament fibroblasts (PDLF) were isolated from periodontal ligament tissue scraped off the middle third of human teeth. For these experiments we used a pool of PDLF from six different patients (3 male, 3 female, age range 17–27 years). Isolation and characterization was performed as previously described [17, 38]. For our experiments, we used PDLF of the 3rd to 6th passage. We determined cell number with a Beckman Coulter Counter Z2™ (Beckman Coulter GmbH, Krefeld, Germany) according to the manufacturer’s instructions.

**Fig. 1:**
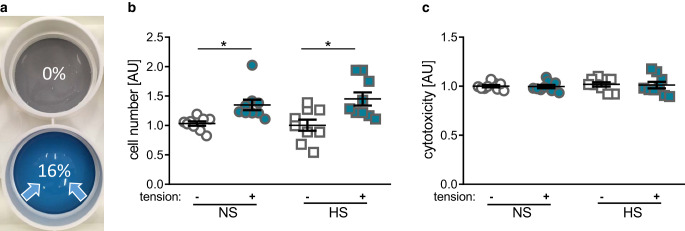
**a** In vitro simulation of tensile strain by isotropic stretching of adherently growing periodontal ligament fibroblasts (PDLF). Determination of cell number (**b**) and cytotoxicity by LDH assay (**c**). *NS* normal salt conditions, *HS* high salt conditions, *AU* arbitrary units, *error bars* error of the mean. *Statistics*: Analysis of variance (ANOVA) using the Games–Howell post hoc test: *p ≤ 0.05 **a** *In-vitro-*Simulation der Zugspannung durch isotrope Dehnung von adhärent wachsenden PDLF (parodontale Ligamentfibroblasten). Bestimmung der Zellzahl (**b**) und der Zytotoxizität durch LDH(Laktatdehydrogenase)-Assay (**c**). (*NS* Normalsalz-, *HS* Hochsalzbedingungen, *AU* arbiträre Einheiten, *Fehlerbalken* Fehler des Mittelwerts. *Statistik*: ANOVA („analysis of variance“) mit Games-Howell-Post-hoc-Test: *p ≤ 0,05)

A total of 70,000 PDLF in 2 ml media (Dulbecco’s Modified Eagle Medium [DMEM] high glucose, D5796, Sigma-Aldrich, Munich, Germany); 10% FBS (fetal bovine serum, P30-3306, PAN-Biotech, Aidenbach, Germany), 1% L‑glutamine (SH30034.01, GE Healthcare Europe, Munich, Germany), 100 µM ascorbic acid (A8960, Sigma-Aldrich, Munich, Germany), and 1% antibiotics/antimycotics (A5955, Sigma-Aldrich, Munich, Germany) were seeded onto 6‑well collagen-I-coated bioflex plates (BF-3001C, Dunn Labortechnik, Asbach, Germany). After 24 h preincubation time without (normal salt [NS] conditions) or with addition of 40 mM NaCl (high salt [HS] conditions, 1162241000, Sigma-Aldrich, Munich, Germany), we performed 16% static isotropic cell stretching for an additional 48 h by using custom-made spherical cap silicone stamps (Fig. 1a) according to an established and published method [30] consisting of two-component silicone (43004,490068, Turbosil, Klasse 4, Dental GmbH, Augsburg, Germany; mixed 1:1 with Dosper evo, Dreve GmbH, Unna, Germany). In a previous study using the same setup, this tensile strain magnitude was shown to be the minimum magnitude to significantly affect the expression of proinflammatory genes in PDLF [30].

### Assessment of cell number

After 48 h of isotropic tensile strain, the medium was removed and PDLF were scraped off the bioflex membrane in 1 ml phosphate buffered saline (PBS) for cell number determination. Cells were counted automatically using a Beckman Coulter Counter Z2™ (Beckman Coulter GmbH, Krefeld, Germany) according to the manufacturer’s instructions.

### LDH cytotoxicity assay

To test for cytotoxicity, we performed lactate dehydrogenase (LDH) assays (04744926001, Roche, Mannheim, Germany) using cell culture supernatants according to the manufacturer’s instructions. A total of 100 µl of cell culture supernatant was mixed with 100 µl freshly prepared LDH solution (consisting of 22 µl catalyst in 1 ml dye). After an incubation period of 30 min at room temperature in the dark, 50 µl stop solution was added and absorbance was measured at 490 nm using an ELISA reader (Multiscan GO Microplate Spectrophotometer, Thermo Fisher Scientific, Waltham, MA, USA), subtracting background absorbance at 690 nm.

### RNA isolation and cDNA synthesis

RNA isolation was performed as previously described according to MIQE (Minimum Information for Publication of Quantitative Real-Time PCR Experiments) guidelines [17, 30]. A total of 500 µl peqGOLD TriFast^TM^ (PEQLAB Biotechnology GmbH, Erlangen, Germany) was added per well and further processed according to the manufacturer’s instructions. The resulting RNA pellet was resuspended in 20 µl nuclease-free doubly distilled water (H_2_O_dd_; T143, Carl Roth, Karlsruhe, Germany). RNA was quantified using a NanoPhotometer (N60; Implen, Munich, Germany). A total of 100 ng RNA per sample was transcribed into cDNA using 1 µl oligo-dT18 primer (SO131, Thermo Fisher Scientific Inc., Waltham, MA, USA), 1 µl random hexamer primer (SO142, Thermo Fisher Scientific Inc.), 1 µl dNTP mix (L785.2, Roti®-Mix PCR3, Carl Roth), 1 µl RNase inhibitor (EO0381, Thermo Fisher Scientific Inc.), 1 µl MLV-reverse transcriptase (M1705, Promega, Fitchburg, WI, USA), 4 µl 5 × M-MLV-buffer (M1705, Promega) in a total volume of 20 µl by addition of nuclease-free H_2_O_dd_ (T143, Carl Roth). Samples were incubated for 60 min at 37 °C and 2 min at 95 °C. Reverse transcription was performed for all samples at the same time to minimize experimental variation.

### Quantitative real-time polymerase chain reaction

Quantitative real-time polymerase chain reaction (RT-qPCR) was performed according to MIQE guidelines [17, 30]. For RT-qPCR the Mastercycler® ep realplex‑S thermocycler (Eppendorf AG, Hamburg, Germany) was used in combination with 96-well PCR plates (TW-MT, 712282, Biozym Scientific GmbH, Hessisch Oldendorf, Germany) and BZO cover sheeting (712350, Biozym Scientific GmbH). Mastermix contained 7.5 µl SYBR®Green JumpStart^TM^ Taq ReadyMix^TM^ (S4438, Sigma-Aldrich, Munich, Germany), 0.75 µl of each primer in a total amount of 13.5 µl by addition of nuclease-free H_2_O_dd_ (T143, Carl Roth, Karlsruhe, Germany) for each sample. Finally 1.5 µl cDNA in duplet per sample was added to the mastermix. RT-qPCR was performed in 45 cycles (initial: 5 min at 95 °C, each cycle: 10 s at 95 °C, 8 s at 60 °C, 8 s at 72 °C). At the end of each extension step SYBR®Green I fluorescence was measured at 521 nm. For calculation of relative gene expression, we used a set of two reference genes (*TBP* and *PPIB*), which have been shown to be stably expressed in PDLF under the conditions investigated [30]. Relative gene expression was calculated as 2^−∆Cq^ with ∆Cq = Cq (target gene) − Cq (geometric mean *TBP/PPIB*) [21]. All primers (Eurofins MWG, Huntsville, AL, USA) are listed in Table 1 and were constructed using NCBI PrimerBLAST according to MIQE guidelines as previously described [17, 19, 30]. For each primer pair and RT-qPCR run, a no-template-control without cDNA was performed. Experiments were repeated two to three times (*N* = 2–3) with at least two to three biological replicates (*n* = 2–3).

**Table 1:** RT-qPCR primer sequences for reference genes (*TBP, PPIB*) and target genes RT-qPCR-Primersequenzen der Referenzgene (*TBP, PPIB*) und der Zielgene

Gene symbol	Gene name	Accession Number (NCBI GenBank)	5′-forward primer-3′ (length/T_m_/%GC/max. ∆G Hairpin &Self-Dimer/Self-Comp./Self-3′-Comp.)	5′-reverse primer-3′ (length/T_m_/%GC/max. ∆G Hairpin &Self-Dimer/Self-Comp./Self-3′-Comp.)
*TBP*	TATA box binding protein	NM_003194.4	CGGCTGTTTAACTTCGCTTCC (21 bp/62.5 °C/52.4%/−0.8/5/0)	TGGGTTATCTTCACACGCCAAG (22 bp/63.4 °C/50.0%/−1.5/3/2)
*PPIB*	Peptidylprolyl isomerase A	NM_000942.4	TTCCATCGTGTAATCAAGGACTTC (24 bp/61.3 °C/41.7%/−1.3/4/2)	GCTCACCGTAGATGCTCTTTC (21 bp/61.2 °C/52.4%/−0.7/4/0)
*ALP*	Alkaline phosphatase	NM_000478.4	ACAAGCACTCCCACTTCATCTG (22 bp/60.3 °C/50.0%/−0.5/3/2)	GGTCCGTCACGTTGTTCCTG (20 bp/61.4 °C/60.0%/−3.3/5/1)
*COL1A2*	Collagen, type I, alpha 2	NM_000089.3	AGAAACACGTCTGGCTAGGAG (21 bp/59.8 °C/52.4%/−3.3/4/2)	GCATGAAGGCAAGTTGGGTAG (21 bp/59.8 °C/52.4%/−2.3/5/0)
*COX‑2*	Cyclooxygenase 2	NM_000963.3	GAGCAGGCAGATGAAATACCAGTC (24 bp/62.7 °C/50.0%/0.0/2/2)	TGTCACCATAGAGTGCTTCCAAC (23 bp/60.6 °C/47.8%/−1.3/4/0)
*FN1*	Fibronectin 1	NM_212482.1	GCCAGTCCTACAACCAGTATTCTC (24 bp/62.7 °C/50.0%/−0.3/4/2)	GCTTGTTCCTCTGGATTGGAAAG (23 bp/60.6 °C/47.8%/−2.5/4/1)
*IL‑6*	Interleukin 6	NM_000600.3	TGGCAGAAAACAACCTGAACC (21 bp/57.9 °C/47.6%/−1.1/3/0)	CCTCAAACTCCAAAAGACCAGTG (23 bp/60.6 °C/47.8%/−0.8/3/3)
*MMP8*	Matrix-metalloproteinase‑8	NM_002424.2	GCTCATTTTGATGCCGAAGAAAC (23 bp/58.9 °C/43.5%/−0.9/3/0)	CCCTGAAAGCATAGTTGGGATAC (23 bp/60.6 °C/47.8%/−2.0/3/2)
*P4HA1*	Prolyl 4-hydroxylase,α I	NM_000917.3	GCTCTCTGGCTATGAAAATCCTG (23 bp/60.6 °C/47.8%/0.0/2/2)	GTGCAAAGTCAAAATGGGGTTC (22 bp/58.4 °C/45.5%/−3.4/4/0)
*OPG*	Osteoprotegerin	NM_002546.3	TGTCTTTGGTCTCCTGCTAACTC (23 bp/60.6 °C/47.8%/0.0/2/0)	CCTGAAGAATGCCTCCTCACAC (22 bp/62.1 °C/54.6%/−0.9/4/0)
*RANKL*	Receptor activator of NF‑κB ligand	NM_003701.3	ATACCCTGATGAAAGGAGGA (20 bp/54.9 °C/45.0%/−1.3/3/0)	GGGGCTCAATCTATATCTCG (20 bp/54.6 °C/50.0%/−0.5/4/2)
*VEGFA*	Vascular endothelial growth factor A	NM_001171623.1	TGCAGACCAAAGAAAGATAGAGC (23 bp/58.9 °C/43.5%/−3.4/4/2)	ACGCTCCAGGACTTATACCG (20 bp/59.4 °C/55.0%/−1.3/5/2)

*T*_*m*_ melting temperature of primer/specific qPCR product (amplicon), *%GC* guanine/cytosine content, *Comp.* Complementarity, *bp* base pair

### Statistical methods

One-way analysis of variance (ANOVA) followed by Games–Howell post hoc tests was performed using GraphPad Prism version 8.0.0 for Windows (GraphPad Software, San Diego, CA, USA). Welch tests were performed in case of heterogeneity of variance. The significance level was set at *p* ≤ 0.05.

## Results

### Impact of tensile strain and sodium chloride on cell number and cytotoxicity

First, we assessed the PDLF number and possible cytotoxic effects after 48 h of tensile strain and 72 h of sodium chloride treatment. Tensile strain significantly increased cell numbers under normal salt treatment (NS, *p* = 0.035), while high salt treatment (HS; *p* = 0.036) with an additional 40 mM NaCl in the medium had no effect on cell number (Fig. 1b). Neither tension treatment nor addition of 40 mM NaCl showed any cytotoxic effects as no significantly increased LDH activity could be detected (*p* = 0.889) in the cell culture supernatant throughout the tested conditions (Fig. 1c).

### Effects of tensile strain and sodium chloride on extracellular-matrix-forming genes

**Fig. 2:**
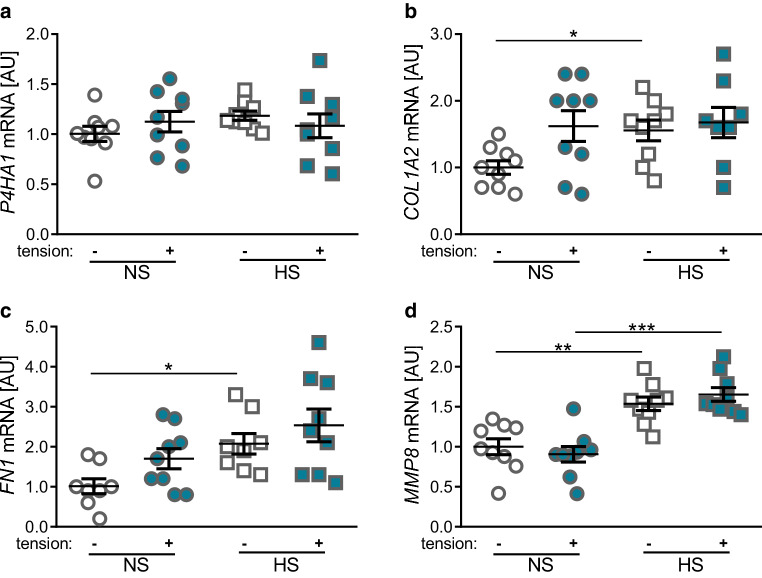
Changes in expression of the extracellular-matrix-reorganizing genes *P4HA1* (**a**), *COL1A2* (**b**), *FN1* (**c**), and *MMP8* (**d**). *NS* normal salt conditions, *HS* high salt conditions, *AU* arbitrary units, *error bars* error of the mean. *Statistics*: Analysis of variance (ANOVA) using the Games-Howell post hoc test: *p ≤ 0.05, **p ≤ 0.01, ***p ≤ 0.001 Veränderungen in der Expression der extrazellulären-Matrix-reorganisierenden Gene *P4HA1* (**a**), *COL1A2* (**b**), *FN1* (**c**) und *MMP8* (**d**). (*NS* Normalsalzbedingungen, *HS* Hochsalzbedingungen, *AU* arbiträre Einheiten, *Fehlerbalken* Fehler des Mittelwerts. *Statistik*: ANOVA („analysis of variance“) mit Games-Howell-Post-hoc-Test: *p ≤ 0,05, **p ≤ 0,01, ***p ≤ 0,001)

Next, we investigated expression of genes involved in remodeling and formation of the extracellular matrix (prolyl-4-hydroxylase‑1 [*P4HA1*], collagen-1-α‑2 [*COL1A2*], fibronectin1 [*FN1*], and matrix-metalloproteinase‑8 [*MMP8*]). Neither tensile strain nor salt treatment showed significant effects on the gene expression of *P4HA1* (*p* = 0.553, Fig. 2a). In contrast, gene expression of *COL1A2 *(*p* = 0.038, Fig. 2b) and *FN1* was significantly elevated under HS conditions (*p* = 0.024, Fig. 2c) without additional tensile strain. Tension had no significant effect on* COL1A2 *or *FN1 *gene expression under NS conditions (*COL1A2*: *p* = 0.120, *FN1*: *p* = 0.173) or under high salt conditions (*COL1A2*: *p* = 0.998, *FN1*: *p* = 0.780). Gene expression of *MMP8* was elevated after addition of 40 mM NaCl without (*p* = 0.004) and with stretching (*p* < 0.001) of PDLF (Fig. 2d). Tensile strain itself had no significant effect on *MMP8* gene expression levels (NS: *p* = 0.904, HS: *p* = 0.769).

### Effects of tensile strain and sodium chloride on angiogenetic and bone-forming genes

**Fig. 3:**
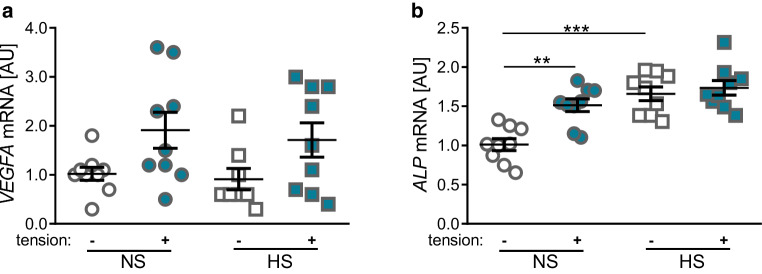
Changes in expression of the angiogenesis-inducing gene *VEGFA* (**a**) and the osteoblastogenesis-inducing gene *ALP* (**b**). *NS* normal salt conditions, *HS* high salt conditions, *AU* arbitrary units, *error bars* error of the mean. *Statistics*: Analysis of variance (ANOVA) using the Games–Howell post hoc test: **p ≤ 0.01, ***p ≤ 0.001 Veränderungen in der Expression des Angiogenese-induzierenden Gens *VEGFA* (**a**) und des Osteoblastogenese-induzierenden Gens *ALP* (**b**). (*NS* Normalsalzbedingungen, *HS* Hochsalzbedingungen, *AU* arbiträre Einheiten, *Fehlerbalken* Fehler des Mittelwerts. *Statistik*: ANOVA („analysis of variance“) mit Games-Howell-Post-hoc-Test: **p ≤ 0,01, ***p ≤ 0,001)

The vascular endothelial growth factor A (*VEGFA*) is involved in tissue neoformation and responsible for growth of blood vessels. Surprisingly, neither tensile strain (NS: *p* = 0.170, HS: *p* = 0.262) nor addition of NaCl (without tension: *p* = 0.972, tension: *p* = 0.979) showed a significant effect on gene expression of *VEGFA *(Fig. 3a), although an increase of *VEGFA* gene expression under tensile strain was observed by tendency. The alkaline phosphatase (*ALP*) gene is involved in bone formation. We observed a significant increase in *ALP* gene expression after tensile strain under NS conditions (*p* = 0.002, Fig. 3b). Treatment of PDLF with 40 mM NaCl also increased *ALP* gene expression significantly (*p* < 0.001). However, we no longer detected an increased *ALP* gene expression due to tensile strain under HS conditions (*p* = 0.312, Fig. 3b).

### Effects of tensile strain and HS on proinflammatory genes

**Fig. 4:**
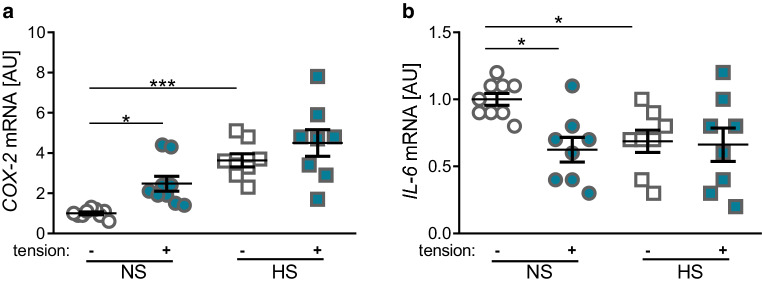
Changes in expression of the proinflammatory genes *COX‑2* (**a**) and *IL‑6* (**b**). *NS* normal salt conditions, *HS* high salt conditions, *AU* arbitrary units, *error bars* error of the mean. *Statistics*: Analysis of variance (ANOVA) using the Games–Howell post hoc test: *p ≤ 0.05, ***p ≤ 0.001 Veränderungen in der Expression der proinflammatorischen Gene *COX‑2* (**a**) und *IL‑6* (**b**). (*NS* Normalsalzbedingungen, *HS* Hochsalzbedingungen, *AU* arbiträre Einheiten, *Fehlerbalken* Fehler des Mittelwerts. *Statistik*: ANOVA („analysis of variance“) mit Games-Howell-Post-hoc-Test: *p ≤ 0,05, ***p ≤ 0,001)

Next we focused on the gene expression of proinflammatory genes like cyclooxygenase 2 (*COX‑2*) and interleukin 6 (*IL‑6*). Tensile strain resulted in a significant increase of *COX‑2 *gene expression (*p* = 0.017, Fig. 4a), whereas *IL‑6* gene expression was significantly reduced (*p* = 0.019, Fig. 4b) under NS conditions. Salt treatment enhanced *COX‑2* gene expression (*p* < 0.001, Fig. 4a) and also reduced *IL‑6* gene expression (*p* = 0.031, Fig. 4b) compared to NS conditions.

### Effects of cell stretching and salt treatment on the *RANKL/OPG* ratio

**Fig. 5:**
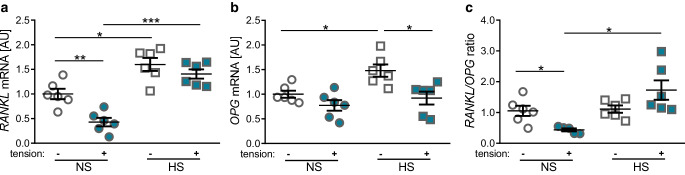
Changes in expression of the bone remodeling genes *RANKL* (**a**) and *OPG* (**b**) as well as *RANKL/OPG* ratio (**c**). *NS* normal salt conditions, *HS* high salt conditions, *AU* arbitrary units, *error bars* error of the mean. *Statistics*: Analysis of variance (ANOVA) using the Games–Howell post hoc test: *p ≤ 0.05, **p ≤ 0.01, ***p ≤ 0.001 Veränderungen der Expression der am Knochenumbau beteiligten Gene *RANKL* (**a**) und *OPG* (**b**) sowie des *RANKL/OPG*-Verhältnisses (**c**). (*NS* Normalsalzbedingungen, *HS* Hochsalzbedingungen, *AU* arbiträre Einheiten, *Fehlerbalken* Fehler des Mittelwerts. *Statistik*: ANOVA („analysis of variance“) mit Games-Howell-Post-hoc-Test: *p ≤ 0,05, **p ≤ 0,01, ***p ≤ 0,001)

The *RANKL/OPG* ratio plays a major regulating role during bone resorption. Tensile strain reduced gene expression of *RANKL* significantly (*p* = 0.008, Fig. 5a), whereas *OPG* gene expression was not affected by PDLF stretching (*p* = 0.355, Fig. 5b). This resulted in a significantly reduced *RANKL/OPG* ratio (*p* = 0.044, Fig. 5c) during tensile strain under NS conditions. HS conditions increased *RANKL* gene expression without (*p* = 0.023) and with additional tensile strain (*p* < 0.001). *OPG* gene expression was also increased under HS conditions without tensile strain (*p* = 0.042, Fig. 5b), whereas *OPG* expression was not affected under HS conditions with tension treatment (*p* = 0.815). This led to a significantly increased *RANKL/OPG* ratio under HS conditions during tensile strain compared to NS conditions with cell stretching (*p* = 0.033, Fig. 5c).

## Discussion

In this study, we investigated the effects of tensile strain and salt (sodium chloride) on the expression levels of genes involved in extracellular matrix reorganization, angiogenesis, bone remodeling, and inflammation in PDLF. We could show that application of tension resulted in a reduced *RANKL/OPG* ratio, which was accompanied by enhanced *ALP* gene expression indicating elevated bone formation. Tensile strain increased *COX‑2*, but concurrently reduced *IL‑6* gene expression. Surprisingly, we detected no effects of tension on genes involved in extracellular matrix remodeling or angiogenesis, whereas salt had a significant impact.

During orthodontic tooth movement, PDLF are suspected to be involved in extracellular matrix remodeling especially in the formation and breakdown of collagen fibrils [25]. For that reason, we analyzed expression of genes involved in collagen formation (*COL1A2, P4HA1*) and degradation (*MMP8*), but also fibronectin 1 (*FN1*), which interacts with collagen and other molecules of the extracellular matrix like heparin sulfate and serves as an adhesion molecule [36]. Furthermore, *FN1* has already been associated before with extracellular matrix remodeling during orthodontic tooth movement [1]. We detected no changes in expression of genes involved in collagen synthesis after application of tension. Howard et al. reported increased FN1 expression after 10% cyclic tensile strain; however, COL1 expression was not changed by PDLF stretching [11], supporting our data. A recent study investigated collagen and fibronectin expression in histological samples. They observed a downregulation of FN1, while COL1 was upregulated at the tension side [26]. Compressive force treatment was reported to affect collagen formation within 24 h because of increased gene expression of *COL1A2* and *P4HA1* [38]. In contrast to genes involved in collagen formation, *FN1* was not affected by compressive force treatment [38]. MMPs are proteolytic enzymes, which degenerate different components of the extracellular matrix [4]. In this study we investigated the gene expression of *MMP8*, which acts as collagenase and is expressed by PDLF [46]. Contrary to our results it was reported that collagenases like MMP8 were upregulated by tensile forces with the strength of the tensile strain playing a crucial role [5, 13, 31], whereas MMP8 was downregulated after compressive force treatment in PDLF [39, 43]. In the current study, salt (sodium chloride) treatment affected gene expression of *COL1A2, FN1* and *MMP8* in PDLF, as reported previously [39]. Salt consumption has already been reported to be involved in extracellular matrix reorganization, as it impacts on glycosaminoglycan sulfatation [45, 49].

Application of orthodontic forces changes the blood flow in the surrounding tissue. To avoid hypoxic conditions, vascular endothelial growth factor A (VEGFA) expression is induced in the periodontal ligament (PDL) due to mechanical strain. VEGFA is a growth factor involved in the reshaping of blood vessels and angiogenesis [7]. Increased VEGFA expression was reported at compression and tension areas of the PDL in histological samples after tooth movement [27]. In this study, however, we detected no significant effect of tensile strain or salt on *VEGFA* gene expression. As increased *VEGFA* gene expression, however, occurred quite early after the onset of mechanical strain [38], our timing of detection might have been too late, as we analyzed *VEGFA* gene expression not earlier than after 48 h of tensile strain.

According to the common pressure–tension theory during orthodontic tooth movement, bone resorption happens at the pressure areas, while bone formation occurs at tension areas of the PDL [25]. Alkaline phosphatase (*ALP*) activity is elevated in the periodontal ligament compared to other connective tissues and is associated with bone formation [10]. In line with our data, static and cyclic tensile strain increased ALP expression dependent on the applied magnitude of tensile strain [12, 30, 31, 50], which may enhance the osteoblastic phenotype of PDLF and prompt bone formation [12, 55]. Furthermore, increased ALP levels were observed in human crevicular fluid after orthodontic treatment [15, 24, 35]. Salt treatment enhanced *ALP* gene expression, suggesting that NaCl promotes an osteoblastic phenotype of PDLF.

PDLF modulate the expression of proinflammatory genes in reaction to orthodontic forces [16, 30, 38]. In this study gene expression of *COX‑2* was increased after 48 h of tensile strain. This was in line with Shimizu et al. who reported that enhanced COX‑2 expression was accompanied by increased PG-E2 levels in PDLF after stimulation with cyclic stretching [40]. As already reported, salt treatment of PDLF also enhanced *COX‑2* gene expression [39]. As it is well established that NaCl increases expression of the osmoprotective transcription factor NFAT5 [23, 33] and that *COX‑2* is an NFAT5 target gene [9], this is not by surprise. In contrast to *COX‑2*, gene expression of *IL‑6* was reduced with tension treatment and NaCl addition. This is in line with prior publications reporting simultaneous increase of *COX‑2* and reduction of *IL‑6* expression upon tensile strain [30, 39]. IL‑6 modulates the extent of immune responses during inflammation [34] and can influence osteoclastogenesis [20]. Reduction of *IL‑6* expression after stretching and NaCl could contribute to the osteoblastic phenotype of PDLF.

Bone metabolism strongly depends on the interaction of RANKL (receptor activator of NF-κB ligand) and OPG (osteoprotegerin) [47]. While binding of RANKL to the RANK receptor on osteoclast precursor cells is critical for osteoclast formation and activation, secretion of the decoy receptor OPG inhibits this interaction [47]. In contrast to pressure application [38, 39], tensile strain resulted in reduced *RANKL* expression, while *OPG* gene expression remained unaffected. This resulted in a shifting of the *RANKL/OPG* ratio towards *OPG* suggesting less bone resorption. As already reported, salt treatment increased gene expression of *RANKL* and *OPG* in PDLF without tensile strain [39]. In contrast to the normal salt-treated PDLFs, we observed a reduction of *OPG* gene expression under high salt treatment with additional tensile strain, resulting in an increased *RANKL/OPG* ratio upon salt treatment with stretching. Therefore salt, that is sodium chloride, may modulate bone metabolism at the tension site as well.

For our in vitro experiments, we used salt concentrations (40 mM) corresponding to the local Na^+^ accumulation measured under high salt diet in the murine mandible including the associated mucosa [39] to maximize transferability of results to the in vivo situation within the PDL and surrounding alveolar bone. The addition of 40 mM NaCl to the cell culture medium did have an impact on the expression of genes involved in extracellular matrix and bone remodeling as well as prostaglandin synthesis supporting previous results [39]. A high salt environment in combination with force application affected the *RANKL/OPG* ratio under tensile strain as well as during compressive force treatment, indicating a stimulating role of salt on osteoclastogenesis and thereby bone resorption [39]. To further investigate the role of salt on orthodontic tooth movement, in vivo experiments with animals receiving low, normal, and high salt diets with and without orthodontic tooth movement are required. Based on our in vitro results, we surmise that increased sodium concentrations due to high salt intake or possibly a local therapeutic injection into the periodontal ligament may accelerate orthodontic tooth movement due to an increase in osteoclastogenesis in pressure areas as well as elevated osteoblastic activity in tensile areas, but this might also have detrimental effects such as periodontal bone loss or dental root resorptions, which needs to be clarified in further in vivo studies.

## Conclusions

- Salt (NaCl) treatment has an impact on extracellular matrix formation, expression of proinflammatory cytokines and bone metabolism during tensile strain in PDLF (periodontal ligament fibroblasts).
- Additional NaCl exposure increased *ALP* (alkaline phosphatase) expression by PDLF and could thereby promote bone formation at tension areas of the PDL (periodontal ligament).
- Excessive salt intake during orthodontic therapy may cause stimulatory effects on periodontal inflammation and bone resorption, possibly leading to increased tooth movement, but also periodontal bone loss and dental root resorptions.
- Tensile strain did not affect expression of genes involved in angiogenesis or extracellular matrix reorganization in PDLFs.
- PDLFs modulate inflammatory responses and bone remodeling in reaction to static tensile strain.
