# Correction to: Effects of sodium chloride on the gene expression profile of periodontal ligament fibroblasts during tensile strain

**DOI:** 10.1007/s00056-021-00362-7

**Published:** 2021-10-25

**Authors:** Agnes Schröder, Joshua Gubernator, Ute Nazet, Gerrit Spanier, Jonathan Jantsch, Peter Proff, Christian Kirschneck

**Affiliations:** 1 Department of Orthodontics, University Hospital Regensburg, Franz-Josef-Strauß-Allee 11, 93053 Regensburg, Germany; 2 Department of Cranio-Maxillo-Facial Surgery, University Hospital Regensburg, Franz-Josef-Strauß-Allee 11, 93053 Regensburg, Germany; 3 Institute of Clinical Microbiology and Hygiene, University Hospital Regensburg, Franz-Josef-Strauß-Allee 11, 93053 Regensburg, Germany

**Correction to:**

**J Orofac Orthop (2020) 81:360**

10.1007/s00056-020-00232-8

The article “Effects of sodium chloride on the gene expression profile of periodontal ligament fibroblasts during tensile strain”, written by Agnes Schröder, Joshua Gubernator, Ute Nazet, Gerrit Spanier, Jonathan Jantsch, Peter Proff and Christian Kirschneck, was originally published Online First without Open Access. After publication in volume 81, issue 5, page 360–370 the author decided to opt for Open Choice and to make the article an Open Access publication. Therefore, the copyright of the article has been changed to © The Author(s) 2020 and the article is forthwith distributed under the terms of the Creative Commons Attribution 4.0 International License, which permits use, sharing, adaptation, distribution and reproduction in any medium or format, as long as you give appropriate credit to the original author(s) and the source, provide a link to the Creative Commons licence, and indicate if changes were made. The images or other third party material in this article are included in the article’s Creative Commons licence, unless indicated otherwise in a credit line to the material. If material is not included in the article’s Creative Commons licence and your intended use is not permitted by statutory regulation or exceeds the permitted use, you will need to obtain permission directly from the copyright holder. To view a copy of this licence, visit http://creativecommons.org/licenses/by/4.0/.

The original article has been corrected.

